# Autophagy in lung disease pathogenesis and therapeutics

**DOI:** 10.1016/j.redox.2014.12.010

**Published:** 2015-01-02

**Authors:** Stefan W. Ryter, Augustine M.K. Choi

**Affiliations:** Joan and Sanford I. Weill Department of Medicine, Weill Cornell Medical College and New York-Presbyterian Hospital, New York, NY, USA

**Keywords:** ALI, acute lung injury, AMPK, 5′-adenosine monophosphate-regulated kinase, ATG, autophagy related protein, CF, cystic fibrosis, CFTR, cystic fibrosis transmembrane conductance regulator, CLP, cecal ligation and puncture, COPD, chronic obstructive pulmonary disease, CS, cigarette smoke, CSE, cigarette smoke extract, ER, endoplasmic reticulum, IPF, idiopathic pulmonary fibrosis, mtb, *Mycobacterium tuberculosis*, mTOR, mechanistic target of rapamycin, mTORC1, mechanistic target of rapamycin complex I, Nrf2, nuclear factor erythroid 2-related factor, NSCLC, non-small cell lung carcinoma, p62*^SQSTM1^*, 62-kDa protein (sequestosome), PAH, pulmonary arterial hypertension, PAS, pre-autophagosomal site, PH, pulmonary hypertension, RIP3, receptor-interacting protein kinase 3, ULK1, uncoordinated 51-like kinase-1, Autophagy, Cigarette smoke, Lung disease, Mitophagy, Reactive oxygen species

## Abstract

Autophagy, a cellular pathway for the degradation of damaged organelles and proteins, has gained increasing importance in human pulmonary diseases, both as a modulator of pathogenesis and as a potential therapeutic target. In this pathway, cytosolic cargos are sequestered into autophagosomes, which are delivered to the lysosomes where they are enzymatically degraded and then recycled as metabolic precursors. Autophagy exerts an important effector function in the regulation of inflammation, and immune system functions. Selective pathways for autophagic degradation of cargoes may have variable significance in disease pathogenesis. Among these, the autophagic clearance of bacteria (xenophagy) may represent a crucial host defense mechanism in the pathogenesis of sepsis and inflammatory diseases. Our recent studies indicate that the autophagic clearance of mitochondria, a potentially protective program, may aggravate the pathogenesis of chronic obstructive pulmonary disease by activating cell death programs. We report similar findings with respect to the autophagic clearance of cilia components, which can contribute to airways dysfunction in chronic lung disease. In certain diseases such as pulmonary hypertension, autophagy may confer protection by modulating proliferation and cell death. In other disorders, such as idiopathic pulmonary fibrosis and cystic fibrosis, impaired autophagy may contribute to pathogenesis. In lung cancer, autophagy has multiple consequences by limiting carcinogenesis, modulating therapeutic effectiveness, and promoting tumor cell survival. In this review we highlight the multiple functions of autophagy and its selective autophagy subtypes that may be of significance to the pathogenesis of human disease, with an emphasis on lung disease and therapeutics.

## Highlights

• Autophagy may impact the pathogenesis of pulmonary diseases.
• Mitophagy may exert deleterious effects in the pathogenesis of COPD.
• Autophagy can exert pleiotropic effects in lung cancer.
• Targeting autophagy may represent a promising therapeutic strategy in human diseases.

## Graphical abstract

**Figure:**
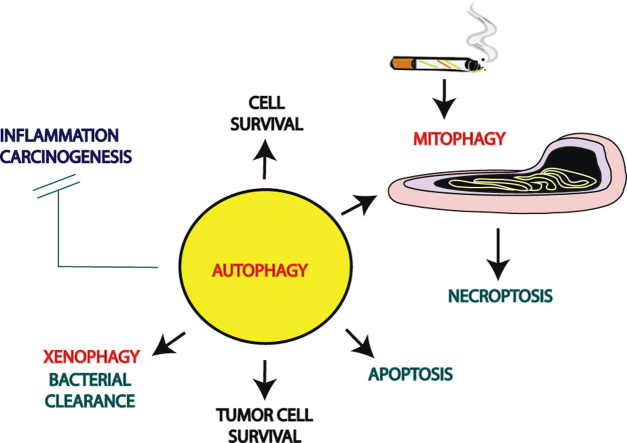

## Introduction

Autophagy is an evolutionarily-conserved cellular program for the turnover of organelles and proteins through a lysosome-dependent degradation pathway [1]. In the most common form of autophagy (also called macroautophagy), cytosolic materials are sequestered into double-membrane compartments called autophagosomes, which subsequently fuse to lysosomes where their contents are enzymatically degraded [2]. Two additional subtypes of autophagy, microautophagy and chaperone-mediated autophagy, have been described elsewhere [3].

Emerging evidence suggests that autophagy exerts complex functions in human diseases that may include both protective and potentially deleterious processes. The known functions of autophagy in the clearance of subcellular debris and in metabolic recycling have led to its general association with detoxification and cellular adaptive or protective mechanisms [1], [4]. Autophagy is now widely recognized as a key regulator of innate and adaptive immune mechanisms, the modulation of which may profoundly impact the pathogenesis of disease [5]. Among those processes affected by autophagy include the regulation of inflammation, antigen presentation, and bacterial clearance [5]. Furthermore autophagy participates in the maintenance of vital organelle populations such as mitochondria, whose dynamic equilibrium is important for cellular bioenergetics and homeostasis [6], [7]. In recent years, it has become evident that the signaling mechanisms that regulate autophagy potentially overlap those that regulate cell death programs such as apoptosis and necroptosis [8], [9], [10], [11]. These observations uncover a potentially deleterious side of autophagy associated with cellular degeneration and cell death, which may have significance in the pathogenesis of disease [8], [9], [10], [11]. Among those diseases where elevated or impaired autophagy has been implicated in the pathogenic process include infectious and inflammatory diseases, metabolic diseases, cancer, neurodegenerative diseases, and diseases of the heart, kidney, liver and lung [12], [13], [14], [15]. The complex actions of autophagy in disease suggests that the autophagy pathway can serve as a therapeutic target to modulate the outcome of disease, which represents an area of rapid current development [2], [13]. In this review, we will focus on the involvement of autophagy specifically in diseases of the lung, with an emphasis on molecular regulation, pathogenesis, and therapeutic targeting.

## Molecular regulation of autophagy

**Fig. 1:**
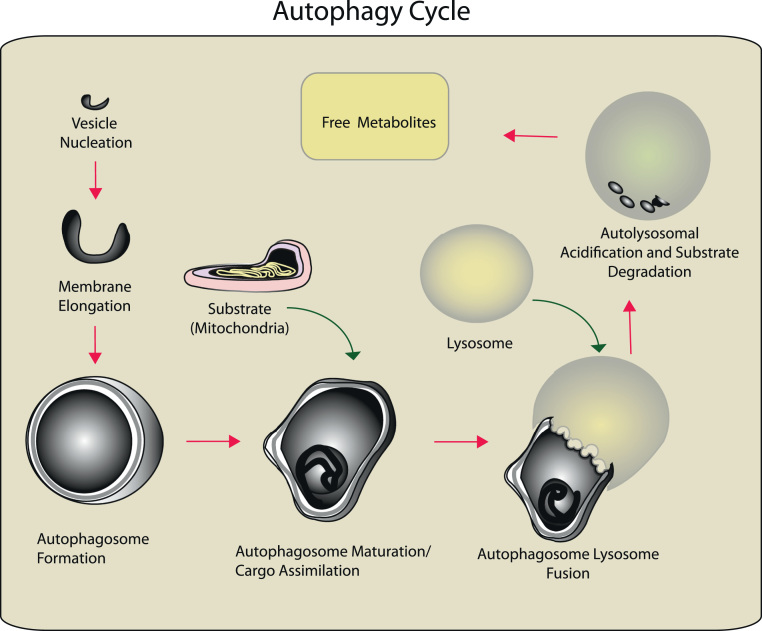
Sequence of the (Macro)-autophagy pathway. Autophagy proceeds through a series of steps that begin with the formation of the isolation membrane at a pre-autophagosomal site. The nascent autophagic membrane elongates to form a double-membrane autophagosome which encompasses a region of cytoplasm, which may include a specific cellular substrate (e.g., damaged mitochondria or aggregated protein). Upon maturation, the autophagosome containing the isolated cargo fuses with the lysosome to form a single-membraned autolysosome. The autophagosomal cargo is then enzymatically degraded in this compartment. The degradation products which may include free amino acids, fatty acids, and nucleotides, are released to the cytoplasm by the action of lysosomal permeases, where they may be reutilized for anabolic pathways.

The process of autophagy involves the intracellular rearrangement of membrane components originating primarily from the endoplasmic reticulum (ER), and other subcellular membranes including the endosome/Golgi system, plasma membrane, mitochondrial membrane, and mitochondria–ER contact sites [16], [17], [18], [19], [20], [21], [22], [23], [24]. Autophagy proceeds through sequential steps that begin with the formation of the phagophore or isolation membrane at a pre-autophagosomal site (PAS) (Fig. 1). The nascent autophagic membrane elongates to form a double-membrane autophagosome which captures a region of cytoplasm, or in the case of selective autophagy, a specific cellular substrate (e.g., damaged mitochondria or aggregated protein). Upon maturation, the autophagosome containing the isolated cargo then fuses with the lysosome to form a single-membrane compartment called the autolysosome. The autophagosomal cargo is then degraded in this compartment by lysosomal acid hydrolases and other degradative enzymes. The resulting degradation products which may include free amino acids, fatty acids, and nucleotides, are released to the cytoplasm by the action of lysosomal permeases, where they may be reutilized for anabolic pathways [1].

**Fig. 2:**
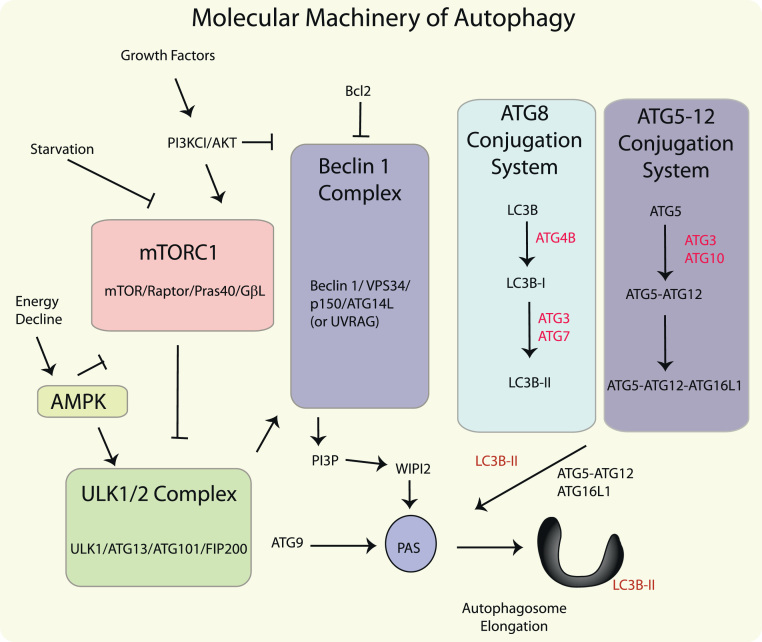
Molecular regulation of autophagy. Autophagy responds to negative regulation by growth factor stimuli that regulate the Class I phosphatidylinositol-3-kinase (PI3K/AKT) pathway, which upregulates the mTOR pathway. mTOR resides in a macromolecular complex (mTORC1): this multi-protein complex is activated by nutrient associated signals including amino acids and growth factors, and negatively regulates autophagy by interacting with the ULK1 complex. Autophagy also responds to regulation by depletion of cellular energy charge through the increased activity of the 5′-adenosine monophosphate (AMP)-activated protein kinase (AMPK). In response to elevated AMP levels, AMPK inactivates mTORC1 and activates ULK1, which can activate Beclin1 and promote the trafficking of mATG9. The initiation of autophagosome formation is also regulated by the autophagy protein Beclin 1 (Atg6). Beclin 1 associates with a macromolecular complex that includes hVps34, a class III phosphatidylinositol-3 kinase (PI3KC3), p150, and ATG14L. The Beclin1 complex produces PI3P which recruits assessory factors in autophagosome formation, including WIPI2. Autophagosome elongation requires two ubiquitin-like conjugation systems, the ATG5-12 conjugation system, and the ATG8 (LC3) conjugation system. Autophagy protein LC3-II remains associated with the maturing autophagosome.

The initiation and execution of autophagy is regulated by a number of autophagy-related (Atg) proteins that were originally identified in yeast. The mammalian homologs (ATG) of these proteins have been identified and constitute the core autophagy machinery [25], [26]. Autophagy responds to regulation by upstream signals initiated by glucose or amino acid starvation, and constitutes an adaptive response under these conditions [1]. The major molecular regulators of autophagy in response to starvation or energy depletion include the mammalian target of rapamycin complex 1 (mTORC1) and the energy sensing, 5′-adenosine monophosphate-regulated kinase (AMPK), respectively (Fig. 2). The mammalian target of rapamycin (mTOR) pathway, which suppresses autophagy during nutrient replete conditions, is activated by growth factors through the Class I phosphatidylinositol-3-kinase (PI3K)/Akt-pathway [27]. In addition to mTOR protein, mTORC1 contains the regulatory-associated protein of mTOR (Raptor), mammalian lethal with SEC13 protein 8 (mLST8), and the 40-kDa proline-rich Akt/PKB substrate (PRAS40) [28]. Inhibition of mTORC1 by starvation or rapamycin results in the activation of autophagy, through de-repression of its substrate complex, the uncoordinated 51-like kinase-1 (ULK1) complex, which consists of ULK1, Atg13, FIP200/RB1CC1, and Atg101 [29], [30], [31], [32], [33]. AMPK, which is upregulated by increasing AMP levels, downregulates mTORC1 by phosphorylating Raptor, and also directly activates ULK1 [34], [35]. Recent studies suggest that AMPK-dependent ULK1 phosphorylation regulates the trafficking of mAtg9, a transmembrane protein responsible for membrane vesicle delivery to the PAS [36].

Autophagy is also regulated by the Beclin1 complex, consisting of Beclin1 (homolog of Atg6), the class III PI3K (PI3KC3/Vps34), p150, and Atg14L or UVRAG [37], [38]. A link between ULK1 and the regulation of Beclin 1 complex through direct phosphorylation of Vps34 has recently been described [39], [40]. Beclin 1 complexes interact with several additional inhibitory (e.g., Bcl-2, Bcl-X_L_) or activating (e.g., Ambra 1) proteins [37]. Activation of the intrinsic PI3KC3 activity of the Beclin 1 complex results in the generation of phosphatidylinositol-3-phosphate (PI3P), which is required for the formation of the autophagosome. PI3P recruits accessory protein factors that include the double FYVE-containing protein-1 (DFCP1) and WD-repeat protein interacting with phosphoinositides (WIPI) proteins, which may exert crucial functions in autophagosome assembly [29], [41], [42]. Recent studies also implicate soluble *N*-ethylmaleimide-sensitive factor attachment protein receptor (SNARE) proteins as crucial determinants in membrane recruitment and autophagosome assembly [43], [44].

The elongation of the isolation membrane to form the mature autophagosome requires two ubiquitin-like conjugation systems: the ATG5–ATG12 conjugation system and the LC3/ATG8 conjugation system [45]. ATG5–ATG12 conjugates, in association with ATG16L1, are required for autophagosome assembly. The ubiquitin-like protein microtubule-associated protein-1 light chain 3 (LC3B) (homolog of yeast Atg8) acts an important mediator of autophagosome formation [26]. The ATG4B endopeptidase cleaves pro-LC3 to generate LC3B-I. In mammals, the conversion of LC3B-I (unconjugated form) to its phosphatidylethanolamine (PE)-conjugated and membrane-associated form LC3B-II represents a crucial step in autophagosome biogenesis [45], [46]. LC3B-II remains incorporated in the autophagosomal membrane until the autophagosome–lysosome fusion step. During the late stages of autophagy, LC3B-II associated with the outer autophagosome membrane is recycled by ATG4B, whereas LC3B-II on the inner membrane is degraded by lysosomal activity [26].

## Selective autophagy

While autophagy can serve as a non-specific degradation system, recent research indicates a high degree of selectivity for specific subcellular targets in processes referred to as “selective autophagy” [47], [48]. The modification of subcellular targets by ubiquitination represents a universal signal for identification of selective autophagy substrates [49]. The selective targeting of autophagy substrates to the autophagosome is assisted by cargo adaptor proteins (e.g., p62*^SQSTM1^*) that can associate both with ubiquitinated substrates and with ATG8 homologs at the autophagosomal membrane through a specialized LIR (LC3-interacting region) [50]. The turnover of mitochondria through selective autophagy pathways is termed *mitophagy*[6], [7]. The classical model for the regulation of mitophagy involves the activation of the mitochondrial kinase PINK1 which recruits the E3: ubiquitin ligase Parkin to the mitochondria where it subsequently ubiquitinates proteins on the outer mitochondrial membrane [6], [7], [51]. Recent studies indicate that PINK1 directly phosphorylates ubiquitin, as a prerequisite for Parkin activation [52], [53]. The capacity of autophagy to clear intracellular pathogens such as bacteria, viruses, and parasites is collectively referred to as *xenophagy*[5], whereas the selective autophagic degradation of protein aggregates is termed *aggrephagy*[54]. We have recently described a novel pathway for the selective autophagic degradation of cilia components, a process we have termed *ciliophagy*[55]. Additional subtypes of selective autophagy and their specific cargo adaptors have been reviewed elsewhere [56].

## Autophagy, oxidative stress, and inflammation

Oxidative cellular stress describes a condition whereby the metabolic production of reactive oxygen species (ROS) supersede cellular antioxidant capacity, leading to damage to cellular macromolecules such as DNA, lipids, and proteins [57]. Oxidative stress is associated with the mitochondrial or enzymatic production of superoxide anion radical (O_2_^−^), and its dismutation product hydrogen peroxide (H_2_O_2_). Production of these species may lead to the generation of additional reactive and deleterious species including the hydroxyl radical and peroxynitrite [58]. The interrelationship between the activation of autophagy as a stress response, and the propagation of cellular injury by oxidative stress has long been proposed [59], [60]. Experimental evidence with model compounds such as H_2_O_2_ and respiratory chain inhibitors suggests that the autophagy can be upregulated in response to oxidative stress [61], [62], [63], [64]. Furthermore, we have demonstrated that autophagy can be activated by increasing or decreasing the ambient oxygen tension [65], [66]. Autophagy may represent a general cellular protective mechanism against oxidative stress, by acting as a degradative pathway for oxidatively-modified substrates, including protein and phospholipids [4], [67]. The function of autophagy in mitochondrial homeostasis may also be of critical importance during oxidative stress, as mitochondria represent an intracellular source of ROS, as well as a functional target for ROS generation [68].

The activation of autophagy by oxidative stress may be triggered by accumulation of damaged substrates. However, limited evidence suggests that components of the autophagy machinery (i.e., ATG4B) may be subjected to direct redox regulation [64]. Furthermore, recent studies suggest a functional cross-talk between the regulation of the mammalian antioxidant response and that of autophagy. Nuclear factor erythroid 2-related factor (Nrf2) serves as a master regulator of cellular antioxidant defenses. Nrf2 dissociates from its cytoplasmic anchor, the Kelch-like ECH-associated protein 1 (Keap-1) and binds to antioxidant response elements in promoters of genes critical for the antioxidant response [69], [70]. The autophagy cargo adaptor protein p62 has recently been identified as an Nrf2-regulated gene [69]. p62 interacts with and promotes the displacement of Keap-1 from Nrf2, and thus activates Nrf2 transcriptional activity [71]. Keap1 is also constitutively degraded by p62-dependent autophagy [72].

Autophagy has been implicated in the regulation of inflammation, which is intimately linked to oxidative stress [56]. We have identified an important function of autophagy in the ROS-dependent regulation of the inflammasome signaling pathway in activated macrophages [73]. Inflammasomes represent an inflammatory signaling platform activated by infection or stress that govern the maturation and secretion of pro-inflammatory cytokines such as IL-1β and IL-18 [74]. Genetic deletion of autophagy proteins (i.e., Beclin 1, LC3B) in primary macrophages caused enhancement of mitochondrial ROS generation, and promoted NOD-like receptor family, pyrin domain containing 3 (NLRP3) inflammasome activation in response to pro-inflammatory stimuli [73], [75]. Similar observations have been made in models of high fat diet-induced metabolic disease, such that palmitate-induced disruption of mitophagy in macrophages was associated with increased activation of the NLRP3 inflammasome [76].

## Autophagy in acute lung injury and sepsis

**Fig. 3:**
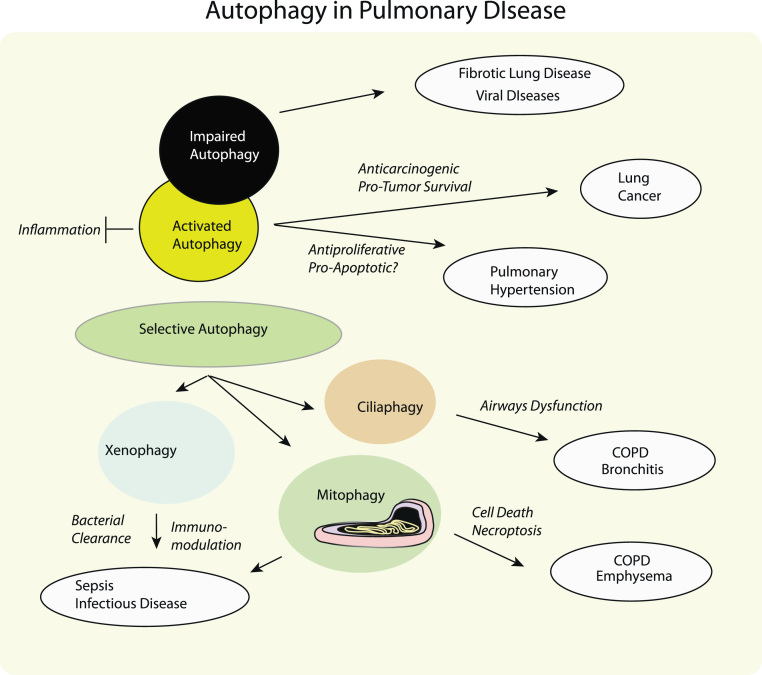
Significance of autophagy in pulmonary disease. Autophagy may exert multiple functions that may be relevant to the pathogenesis of lung disease. These include the general protective effects of autophagy in metabolic recycling, the regulation of inflammation, and the regulation of cell death pathways. The protective aspects of autophagy against carcinogenesis in primary cells, may also provide a paradoxical survival advantage to growing tumors. In certain diseases such as fibrotic lung diseases, impaired autophagy may influence the pathogenesis. Specialized subtypes of selective autophagy may gain importance in select pulmonary disorders. The xenophagy function of autophagy may be important in infectious diseases and sepsis. The mitophagy and ciliaphagy programs have recently been implicated in the pathogenesis of chronic lung disease.

Acute lung injury (ALI) and sepsis continue to represent primary causes of morbidity and mortality during the management of critically ill patients. Although relatively few studies have been performed in this area, recent model studies from our group and others have implicated autophagy as potentially important in the pathogenesis of both ALI and sepsis [65], [77], [79] (Fig. 3).

High oxygen therapy (hyperoxia) is used in critical care settings to maintain tissue oxygenation. ALI may occur as the result of mechanical ventilation or hyperoxia treatments, the latter which causes enhanced ROS production. Extended exposure to hyperoxia (>95% O_2_) is a highly reproducible model of ALI in mice, which targets the pulmonary epithelium [78]. We have shown that hyperoxia exposure causes elevation of histological and biochemical markers of autophagy in vivo, including autophagosome formation and LC3B-II accumulation [65]. Studies of cultured pulmonary epithelial cells, a primary target of hyperoxia in the lung, also indicated that hyperoxia can activate LC3B conversion in vitro, which was reversible by antioxidants [65]. Genetic interference of the autophagosome-associated protein LC3B sensitized epithelial cells to hyperoxia induced cell death and augmented the extrinsic apoptosis pathway [65]. These results suggested that LC3B acts as a pro-survival factor in oxygen-dependent cytotoxicity, and underscore a potential cross-talk between autophagy and apoptosis during oxygen toxicity [65].

We have recently demonstrated that mice subjected to cecal ligation and puncture (CLP), a model of polymicrobial sepsis, display evidence for elevated autophagy in the lung tissue, including increased LC3-II expression and accumulation of autophagosomes [79]. Becn1^+/−^ mice were susceptible to the lethal effects of CLP. Becn1^+/−^ mice displayed reduced bacterial clearance from the blood and vital organs subsequent to CLP. Furthermore, Beclin 1 was required for the therapeutic effectiveness of carbon monoxide, a candidate anti-inflammatory therapy, at alleviating mortality and promoting bacterial clearance in this model [79]. We concluded that Beclin 1 can significantly contribute to sepsis survival by enhancing bacterial clearance [79]. Additional studies in the *Staphylococcus aureus* sepsis model are suggestive of increased mitophagy as a component of lung responses to inflammation. The study noted decreased Beclin 1 protein and increased p62 accumulation, suggestive of dysregulated autophagy, but observed increased in LC3-II accumulation that colocalized with mitochondria, suggestive of activated mitophagy [80].

## Autophagy in infectious lung disease

An emerging role for autophagy as a central player in innate and adaptive immune functions has recently emerged [5]. Autophagy makes an important contribution to host defense against various microbes including bacteria, viruses, and parasites [5], [12], [13]. The anti-bacterial and anti-pathogenic functions of autophagy have been widely demonstrated [81], [82]. Phagocytosis of nonpathogenic mycobacteria by macrophages promotes autophagy and apoptosis, which results in the elimination of the pathogen. However, phagocytosis of pathogenic mycobacteria can inhibit the autophagy pathway [83]. Although of potential importance in many inflammatory and infectious diseases, the relevance of autophagy in respiratory infections will be discussed here.

Tuberculosis, the result of infection with the pathogen *Mycobacterium tuberculosis* (Mtb), is a major contributor to global disease burden [84]. During Mtb infection, the mycobacteria remains and replicates in immature phagosomes. Mtb employs a strategy for survival that involves interference with the fusion between phagosomal compartments containing Mtb and lysosomes [85]. In addition, instead of stimulating macrophage apoptosis, phagocytosis of Mtb promotes necrotic cell death, which promotes bacteria dispersal to uninfected cells. As a result, reduced mycobacterial antigen presentation and chronic Mtb infection occur [86].

Therapeutic upregulation of autophagy can reduce intracellular replication and survival of Mtb [81], [82], [86], [87], [88]. Several therapeutics that stimulate autophagy through mTORC1 inhibition have been recently shown to be effective against Mtb infection [89], [90]. Conversely, chemical inhibitors of autophagy promote Mtb infection [82].

Autophagic process may assist in the generation of anti-virulence factors against Mtb, through degradation of substrate proteins [91], [92]. Interferon-gamma (IFN-γ) production acts as an important host defense factor against Mtb*.* Macrophages stimulated with IFN-γ induce autophagy, and this response facilitates the resolution of infection [82], [91]. IFN-γ stimulation can thereby bypass the inhibition of lysosomal fusion of virus containing phagosomes, leading to the degradation of the bacteria by p62-dependent selective autophagy [91], and resolution of infection. IFN-γ induced autophagy requires the p47 guanosine triphosphatase IRGM-1 [82], [93], [94]. Small nucleotide polymorphisms occurring in the IRGM-1 gene have been linked to increased susceptibility to Mtb infection [95].

Recent studies have identified a selective autophagy pathway for Mtb processing [96]. The bacterial early secretory antigenic target 6 (ESAT-6) system 1 (ESX-1) secretion system mediates phagosomal permeabilization to permit the ubiquitin-mediated autophagy pathway access to phagosomal Mtb. The stimulator of interferon genes (STING)-dependent cytosolic pathway recognizes extracellular bacterial DNA and tags bacteria with ubiquitin. Autophagy cargo adaptors, p62 and NDP52, subsequently recognize ubiquitinated Mtb and target them to autophagosomes.

Autophagy has also been described as a defense mechanism against other respiratory pathogens. Genetic deficiency of *Atg9* promoted the growth of *Legionella pneumophila*, the causative agent in Legionnaire’s disease, which suggests a role of autophagy in defense against this organism [97]. Genetic deficiency of *Atg7* sensitized mice to the lethal effects of *Klebsiella pneumoniae* infection, involving increased bacterial counts, inflammation, and lung injury [98]. Infection with influenza viruses (e.g., influenza-A) can promote the induction of autophagy, and autophagosome formation which is required for viral replication [99]. Influenza-A proteins (e.g., M2 protein) may also inhibit autophagosome maturation and fusion to the lysosome [100]. In the context of influenza infections, recent studies have uncovered a new role for autophagy in the maintenance of memory B cells, which are required for secondary antibody responses. Mice with B cell-specific deletion of *Atg7* displayed impaired secondary antibody responses, and thereby higher sensitivity to influenza virus challenge [101]. In conclusion, the development of therapeutic strategies involving the modulation of the autophagy pathway to reduce infection and promote adaptive immunity to infectious pathogens may be of considerable interest.

## Autophagy in pulmonary vascular disease

Pulmonary arterial hypertension (PAH) is a serious disease affecting the pulmonary vasculature, that is characterized by sustained elevation of pulmonary arterial pressure (>25 mm Hg at rest) [102]. PAH is classified as a pulmonary-selective vascular remodeling disease in which vascular smooth muscle cells display a proliferative and anti-apoptotic phenotype [102]. Pulmonary arterial remodeling occludes the vessel lumen that leads to right ventricular failure and premature death [103]. We have recently demonstrated an elevated incidence of autophagy in lung tissue derived from patients with various forms of pulmonary hypertension (PH), including PAH [66].

Pulmonary hypertension (PH) is a progressive and often fatal complication of chronic lung disease [104]. Chronic hypoxia induces pulmonary arterial vascular smooth muscle cell proliferation, which is a cause of vascular remodeling during PH [105]. In the mouse model, chronic exposure to hypoxia caused increased incidence of PH which was associated with increased autophagosome formation in lung tissue. Autophagy deficient, LC3B null (Map1lc3B^−/−^) mice displayed heightened indices of PH after exposure to chronic hypoxia compared to wild-type mice [66]. Furthermore, blockade of mTORC1, which induces autophagy, was shown to exert anti-proliferative effects on pulmonary vascular cells [106], [107]. These results, taken together, suggest that stimulation of autophagy or inhibition of the mTOR pathway may have protective effects during the pathogenesis of PH.

In contrast, autophagy deficiency through the knockdown of the autophagy protein Beclin 1 resulted in improved angiogenesis in pulmonary artery endothelial cells from fetal lambs with persistent pulmonary hypertension [108]. We have also demonstrated increased angiogenesis in Beclin1 heterozygous knockout mice subjected to hypoxia [109]. These results suggested that Beclin 1-dependent autophagy may contribute to the pathogenesis of PH. In contrast, chloroquine, an inhibitor of autophagy, has been reported to prevent progression of experimental PH [110]. The LC3 and mTOR pathway have recently been highlighted as potential therapeutic targets in hypoxia-induced PH [111], [112]. Due to conflicting studies, the contribution of autophagy to the pathogenesis of PH and related vascular disorders requires further investigation.

## Autophagy in idiopathic pulmonary fibrosis

Fibrosis is characterized by the excessive extracellular matrix protein deposition in the basement membrane and interstitial tissue of an injured epithelium and expansion of activated mesenchymal cells (*i.e.,* myofibroblasts) [113]. Lung fibroblasts are important components of the interstitium which are principal producers of extracellular matrix as well as participate in wound healing. Defective fibroblast autophagic processes have been implicated in the pathogenesis of IPF. Lung tissues from IPF patients and human lung fibroblasts treated with TGF-β demonstrate increased cellular senescence and decreased autophagic activity as characterized by decreased LC3B protein expression [114], [115]. TGF-β1 inhibits autophagy in human lung fibroblasts. Genetic deletion of the autophagy proteins, LC3B or Beclin 1, potentiated the TGF-β1-induced expression of fibronectin and the myofibroblast marker α-smooth muscle actin in fibroblasts [115]. Treatment of mice with the mTOR inhibitor rapamycin, partially protected against lung fibrosis [115]. Loss of autophagy in patients with IPF may potentiate the effects of TGF-β1 with respect to extracellular matrix production and transformation to a myofibroblast phenotype. In a murine bleomycin model of pulmonary fibrosis, blockade of IL-17A in the lung was shown to protect against fibrosis in part by restoring autophagy [116]. Further research is needed to determine the relationships between autophagy and the molecular mechanisms of fibrogenesis.

## Autophagy in cystic fibrosis

Cystic fibrosis (CF) is a fatal autosomal recessive disease which is caused by mutation in the gene encoding the cystic fibrosis transmembrane conductance regulator (CFTR). CF is characterized by accumulation of hyperviscous mucous, which obstructs the airways, resulting in recurrent pulmonary infections. The most common CFTR mutation is a deletion of phenylalanine at position 508 (CFTR^F508del^) in the CFTR gene [117]. Recent studies have implicated CF as a disease involving impaired autophagy. Cells with CFTRF^508del^ display accumulated polyubiquitinated proteins, defective autophagy and the decreased clearance of aggresomes [118]. Dysfunctional autophagosome clearance in CF has also been shown to contribute to heightened inflammatory responses [119]. Defective CFTR also results in increased ROS production and upregulation of tissue transglutaminase [120]. These events were associated with the crosslinking and inactivation of Beclin 1, leading to sequestration of PI3KC3 and accumulation of p62 [120]. Restoration of Beclin 1 or depletion of p62 rescued the trafficking of mutant CFTR to the cell surface [120]. Genetic targeting of p62 was also recently shown to improve the therapeutic effect of CFTR channel activators [121].

The autophagic clearance of bacteria (xenophagy) may be important in defense against the secondary infections associated with CF. Administration of the mTOR inhibitor rapamycin decreases *Burkholderia cenocepacia* infection and reduces inflammation in the lungs of CF mice [122]. Pharmacological enhancement of autophagy in vivo also effectively promoted bacterial clearance of *Pseudomonas aeruginosa* from the lung [123]. In conclusion, the impairment of autophagy, and several of its selective autophagy subtypes may accelerate the pathogenesis of CF. Functions of autophagy that may be compromised in CF include bacterial clearance, protein aggregate processing, and the maintenance of mitochondria. Thus, strategies aimed at the restoration of autophagy may have therapeutic potential in CF [124].

## Autophagy and mitophagy impact the pathogenesis of chronic obstructive pulmonary disease

Chronic obstructive pulmonary disease (COPD), contributes significantly to the global burden of disease [125]. COPD includes clinical phenotypes of emphysema (loss of alveolar surface area) and bronchitis associated with mucus obstruction of the airways [126]. Cigarette smoke (CS) is the most common risk factor for COPD [127]. The mechanisms underlying the pathogenesis of COPD remain incompletely understood but implicate aberrant inflammatory and cellular responses in the lung, lung vasculature, and airways in response to CS [126], [127]. Our recent studies have evaluated the incidence of autophagy in the pathogenesis of COPD by examining static autophagic markers in human lung tissue of COPD patients. In COPD lung tissues we found an elevation of general autophagy markers, such as the increased expression of the autophagosomal marker LC3-II, and other ATG proteins, and the increased occurrence of autophagosomes in situ [128]. To investigate the involvement of autophagy in the pathogenesis of emphysema, we employed a model of emphysematous airspace enlargement in mice subjected to chronic (6 months) CS exposure. Lung tissue derived from mice chronically exposed to CS displayed increased autophagosome numbers and increased expression of autophagy proteins [128], [129]. Autophagy-deficient LC3B-null mice (Map1lc3B^−/−^) were resistant to CS-induced airspace enlargement during chronic CS exposure [129]. To investigate the involvement of autophagy in the pathogenesis of bronchitis associated with COPD, we employed a mouse model of mucociliary clearance disruption in mice subjected to acute (3 weeks) CS exposure. We found that autophagy-deficient Map1lc3B^−/−^ or Becn1^+/−^ mice were resistant to mucociliary clearance disruption in the airways after subchronic CS exposure in vivo [55].

We have also identified a functional role for autophagic proteins in CS-induced epithelial cell death [128], [129]. In vitro studies with cultured epithelial cells subjected to aqueous cigarette smoke extract (CSE) responded with increased autophagosome formation and accumulation of LC3B-II. CSE induced the extrinsic apoptosis pathway in epithelial cells and downstream activation of pro-apoptotic caspases. Genetic deletion of crucial autophagy proteins (i.e., Beclin 1 or LC3B) inhibited apoptosis in response to CSE exposure in vitro, suggesting that increased autophagy occurred in association with epithelial cell death [128], [129], [130]. In mechanistic studies, we described a potential cross-talk between LC3B and the activation of extrinsic apoptosis in epithelial cells exposed to CSE [129]. Following CSE stimulation, LC3B interacted with the death receptor Fas, a component of the extrinsic apoptosis pathway, and the lipid raft scaffold protein caveolin-1. These data underscore complex interactions between the regulation of autophagy and programmed cell death during CS exposure [129]. In conclusion, autophagy modulation can be observed in lung macrophages, bronchial and epithelial cells upon CS exposure and in the lungs of COPD patients [128], [129], [130]. Our recent studies suggest that autophagy promotes lung epithelial cell death, airway dysfunction, and emphysema in response to CS exposure in vivo [55], [128], [129]. However, the underlying mechanisms remain to be elaborated.

In addition to the general autophagy pathway as we have described, emerging studies suggest that selective autophagy may be important in COPD pathogenesis. Increased accumulation of p62 and ubiquitinated proteins have been detected in lung homogenates from COPD patients and in human bronchial epithelial cells exposed to CSE [131]. CS exposure may impair the delivery of bacteria to lysosomes suggesting that defective xenophagy in alveolar macrophages of smokers may contribute to recurrent infections [132]. Consistent with a detrimental role for the activation of autophagy in chronic lung disease we have also recently described a pro-pathogenic role for selective autophagic processes in the pathogenesis of COPD [55], [133].

We reported that ciliophagy, the selective autophagic degradation of cilia, regulates cilia length during CS exposure [55]. Impaired airway clearance caused by cilia shortening, prevents the elimination of pathogens from the airways and may cause recurrent respiratory infections. We demonstrated that autophagy-deficient (Becn1^+/−^ or Map1lc3B^−/−^) mice, as well as airway epithelial cells isolated from these mice, resisted CS-induced cilia shortening. We identified the cytosolic deacetylase HDAC6 as a signaling mediator of autophagy-mediated cilia shortening during CS exposure [55]. In summary, suppression of the autophagy pathway in vitro and in vivo improved cilia phenotypes and airway function during CS exposure.

Recently, we have also shown that CS exposure in epithelial cells promotes the autophagy-dependent turnover of mitochondria (mitophagy). In cultured pulmonary epithelial cells, we found that CS caused mitochondrial dysfunction associated with a decline of mitochondrial membrane potential, and increased mitochondrial ROS production [133]. CS induced the mitophagy program through the stabilization of the mitophagy regulator PINK1. Genetic deficiency of PINK1 protected against CS-induced cell death and mitochondrial dysfunction in vitro. We have also observed in this model that activation of the mitophagy program can regulate necroptosis, a form of programmed necrosis [133]. Genetic deficiency of PINK1 reduced the phosphorylation of the mixed lineage kinase domain-like protein (MLKL), a substrate for the receptor-interacting protein kinase 3 (RIP3) in the necroptosis pathway. The mitochondrial division/mitophagy inhibitor Mdivi-1 also protected against epithelial cell death in vitro. CS induced epithelial cell death was reduced by administration of chemical inhibitors of necrosis or necroptosis. Mitophagy-deficient Pink1^−/−^ mice were protected against mitochondrial dysfunction, airspace enlargement, and mucociliary clearance disruption during CS exposure in vivo. Inhibition of mitophagy by Mdivi-1 treatment also improved airway phenotypes in CS-exposed mice in vivo. In human COPD, lung epithelial cells displayed increased expression of PINK1 and RIP3. These findings, taken together, implicate mitophagy in acute airways dysfunction and in lung emphysematous changes in response to CS exposure, suggesting that this pathway may represent a therapeutic target for COPD [133].

Mitophagy may contribute to mitochondrial quality control and thereby has been predicted to exert a pro-survival role in oxidative stress [68], [134], [135]. In the specific case of CS exposure, a complex model of toxicant exposure, our results suggest that the activation of mitophagy may promote the induction of necroptosis, a cell death program, and also potentially lead to depletion of the functional mitochondrial pool in the chronic setting [133]. However, the precise mechanisms by which mitophagy can serve to promote tissue injury in the CS exposure model remains unclear. Further studies will be necessary to improve the understanding of the role of mitophagy in the pathogenesis of COPD.

## Autophagy, a multifaceted modulator of carcinogenesis and lung cancer

Autophagy has been recognized as having a complex impact on the initiation, progression, and treatment of cancer. As a crucial component of cellular defense mechanisms, autophagy has a putative anti-carcinogenic effect through the preservation of mitochondria, the clearance of subcellular debris, the recycling of metabolic precursors, and the dampening of inflammation, which can contribute to genetic instability [136]. Paradoxically, the pro-survival effects of autophagy may provide a mechanism that promotes tumor cell survival in established tumors under adverse conditions, and afford tumor cell resistance to chemotherapeutics, thus promoting the progression of cancer. In contrast, autophagy has also been shown to potentiate the lethal effect of chemotherapeutics through mechanisms dependent on autophagy-associated cell death [136]. Monoallelic disruption of the *Becn1* on chromosome 17q21 occurs in human tumors [13]. Abnormal expression of Beclin 1 in tumor tissue is correlated with poor prognosis and aggressive tumor phenotypes [12], [13].

To date, few studies have explored the impact of autophagy specifically in lung cancer and therapeutics. Beclin-1 expression was inversely correlated with tumor size and primary tumor stage in human lung adenocarcinomas and was reduced in non-small cell lung carcinoma (NSCLC) relative to normal tissue [137], [138]. Induction of autophagy through mTOR inhibitors has been associated with radiosensitization in NSCLC cells [139]. The autophagy inhibitor hydroxychloroquine has been tested for general therapeutic efficacy in NSCLC [14]. Genetic deletion of the autophagy protein ATG5 results in impaired progression of KRas(G12D)-driven lung cancer and promotes the survival of tumor bearing mice. However, the initiation of KRas(G12D)-driven lung tumors in these mice was accelerated by ATG5 deletion, emphasizing that autophagy may prevent oncogenesis, but promote tumor growth [140].

## Conclusions and therapeutic implications

It is now clear that autophagy has been associated with pro- and anti-pathogenic effects in human disease. The potential for advantageous and deleterious effects of this process in illustrated in cancer, whereby autophagy can protect against the early stages of carcinogenesis, yet accelerate tumor growth. In certain diseases such as sepsis, autophagy may provide a pro-survival advantage by promoting bacterial clearance, and by modulating inflammation. In cigarette smoke exposure models, we have consistently found an amplification of disease process when autophagy or mitophagy is activated. To date the arsenal of autophagy modulating therapeutics is mostly limited to experimental compounds. Currently, only a few compounds that can modulate autophagy have been evaluated for clinical use, including the mTOR inhibitor rapamycin (an inducer of autophagy) and chloroquine or hydroxychloroquine (inhibitors of autophagy). The effectiveness of these compounds in a given setting may not necessarily exclude off-target effects unrelated to the autophagy pathway. Additional candidate therapeutic compounds that modulate autophagy have been described, including Tat-Beclin 1 peptide [141], inhibitors of histone deacetylases, activators of the AMPK pathway, and Vitamin D [13], [14], [142]. The multifaceted effects of autophagy suggest that a complete understanding of the pathogenic process and the impact of autophagy would be prudent before attempting to modulate autophagy in the context of human disease. Furthermore, a better understanding of the multiple pathways of selective autophagy and their impact on disease pathogenesis may also facilitate the design of more specific therapies for the treatment of pulmonary diseases, and other related diseases where autophagy may contribute to pathogenesis.
